# Statement of Retraction: The LOXL1 antisense RNA 1 (LOXL1-AS1)/microRNA-423-5p (miR-423-5p)/ectodermal-neural cortex 1 (ENC1) axis promotes cervical cancer through the mitogen-activated protein kinase (MEK)/extracellular signal-regulated kinase (ERK) pathway

**DOI:** 10.1080/21655979.2025.2496006

**Published:** 2025-04-22

We, the journal and Publisher of *Bioengineered*, have retracted the following article:

Ping Zhang, Fang Zhao, Ke Jia & Xiaoli Liu (2022) The LOXL1 antisense RNA 1 (LOXL1-AS1)/microRNA-423-5p (miR-423-5p)/ectodermal-neural cortex 1 (ENC1) axis promotes cervical cancer through the mitogen-activated protein kinase (MEK)/extracellular signal-regulated kinase (ERK) pathway, Bioengineered, 13:2, 2567-2584, DOI: 10.1080/21655979.2021.2018975

Following publication, the authors reached out in 2024 to inform the journal that they could not verify the data within the article. They stated that some of the experiments within the article were assisted by a third party and that the reported results could no longer be relied upon. The use of a third party to perform experiments was not disclosed within the article.

As we cannot verify the validity of the published work or that it complied with our editorial policies, we are therefore retracting the article. The corresponding author listed in this publication has been informed.

We have been informed in our decision-making by our editorial policies and the COPE guidelines. The retracted article will remain online to maintain the scholarly record, but it will be digitally watermarked on each page as ‘Retracted’. 

